# Predictive Sub-items and Cutoff Scores of the Functional Assessment for Control of Trunk (FACT) for Gait Independence in Patients with Acute Stroke

**DOI:** 10.1298/ptr.25-E10384

**Published:** 2026-03-06

**Authors:** Hiroto MASHIKO, Daiki KATO, Aki KANEKO, Hwa YOUNG, Rika IKEZAWA

**Affiliations:** 1 Department of Rehabilitation, Nasu Red Cross Hospital, Japan; 2 Medical Management, Department of Public Health, Tohoku University School of Medicine, Japan

**Keywords:** Stroke, Trunk control, FACT, Acute phase, Gait independence

## Abstract

**Objectives:**

In this study, we aimed to identify the predictive subitems and cutoff values of the Functional Assessment for Control of the Trunk (FACT) for independent gait in patients with acute stroke.

**Methods:**

This retrospective observational study included 110 stroke patients. The FACT was assessed early after mobilization. Logistic regression analysis was conducted with independent gait at discharge as the dependent variable and 11 independent variables, including FACT subitems 3–10 and potential confounding factors. Receiver-operating characteristic (ROC) analysis was used to determine cutoff values for the total FACT score in predicting independent gait.

**Results:**

For level-ground gait, FACT items 4 and 5 (odds ratios: 4.71 and 2.89, respectively) were significant predictors. For uneven-ground gait, FACT item 8 (odds ratio: 1.87) was significant. The cutoff values were 11 points (sensitivity: 84.0%; specificity: 93.3%; area under the curve [AUC]: 0.95) for level-ground gait and 15 points (sensitivity: 77.8%; specificity: 95.0%; AUC: 0.89) for uneven-ground gait.

**Conclusions:**

Early FACT scores predicted independent gait. For level-ground gait, coordinated movement of the lower limbs and trunk during forward and lateral weight shifts and extensive lateral weight-shifting ability (items 4 and 5) were key. For uneven-ground gait, the rotational ability of the bilateral lower trunk during extensive lateral weight shifts (item 8) was crucial. The present findings suggest that these predictors and cutoff values can assist in guiding the prioritization of trunk assessment and devising tailored intervention plans aimed at functional gait acquisition during the early physical therapy phase following stroke onset.

## Introduction

Gait impairment is a common disability following a stroke. Trunk control impairment significantly influences gait^1–4)^ and has been reported to be a more critical predictor of gait acquisition than neurological severity^5,6)^ or paretic limb function^6)^. Furthermore, trunk control is a significant predictor of community mobility and social participation after discharge, including hobbies and work^7)^.

Primary measures for assessing trunk function in stroke patients include the Trunk Control Test (TCT), which is used internationally^8,9)^, and the Trunk Impairment Scale (TIS) developed by Verheyden et al.^10)^, which comprises items assessing static sitting balance, dynamic sitting balance, and coordination. In Japan, the trunk subitem of the Stroke Impairment Assessment Set (SIAS) has been reported^11)^. Another tool, the Functional Assessment for Control of the Trunk (FACT), was also developed in Japan^12)^. Similar to the TIS, this assessment is performed in a sitting position, and an English version has been developed^13)^.

The FACT evaluates sitting trunk control through both static and dynamic tasks. Its distinctive feature is that it assesses trunk control in relation to voluntary limb movements incorporated into certain items, rather than evaluating the trunk in isolation^12)^. Items were selected from movements commonly used by physical therapist (PT) in routine assessment and treatment^12)^ and comprise elements that influence gait^14)^, making FACT a treatment-oriented assessment method^12)^ applicable to exercise therapy^15)^. Its measurement properties, including reliability^12)^, validity^14–16)^, and responsiveness^17)^, have been verified. Among its subitems, items 4–8 have been reported as particularly important in assessing trunk control impairment^16)^. Regarding the interpretability of the total score, cutoff values for gait have been reported in subacute cases: 9 points for supervised walking in a cross-sectional study with a mean of 110 days post-stroke^15)^, and 8 points at admission predicting independent gait at discharge in a longitudinal study with a median of 16 days post-stroke^18)^.

However, the specific FACT subitems that influence gait ability and the predictive cutoff values for acute-phase patients remain unclear. In clinical practice, determining the priority of localized trunk control assessment using a standardized scale for gait acquisition often lacks clear indicators and frequently relies on therapists' experience and subjective judgment.

In the clinical setting for patients with acute stroke, where there are cases of discharge or transfer within 2–3 weeks^6)^ of onset and limited time, we considered the necessity of a 2-tiered analysis of the FACT—focusing on both “specific subitems” and the “total score”—as a strategy to efficiently address these challenges. Specifically, the identification of specific subitems provides the rationale for determining the “content” of an intervention by pinpointing “which motor functions” to focus on for patients identified through screening. On the other hand, the cutoff value based on the total score serves as a practical tool for “screening” the potential for independent gait within a limited assessment time, enabling rapid judgment regarding the “necessity” and “priority” of interventions targeting trunk function. In this way, the 2 indicators have a complementary relationship, each supporting different stages of clinical decision-making—“identifying targets” and “screening.” Integrating them was considered to make it possible to stratify the prioritization of assessment and intervention, which has often relied on clinical experience, based on evidence.

Therefore, this study aimed to clarify the following 2 points to facilitate the planning of efficient physical therapy aimed at gait acquisition in patients with acute stroke: first, to identify the FACT subitems assessed early after mobilization that influence independent gait at discharge, thereby providing a basis for determining intervention content; and second, to establish cutoff values for the total FACT score assessed early after mobilization to predict independent gait, thereby providing a screening indicator.

## Methods

### Subjects

This single-center retrospective observational study initially included 292 patients admitted to the neurosurgery ward of Nasu Red Cross Hospital (our institute), an acute care hospital, with a diagnosis of cerebral infarction or hemorrhage between April 2023 and December 2024, who were referred for rehabilitation.

**Fig. 1.**
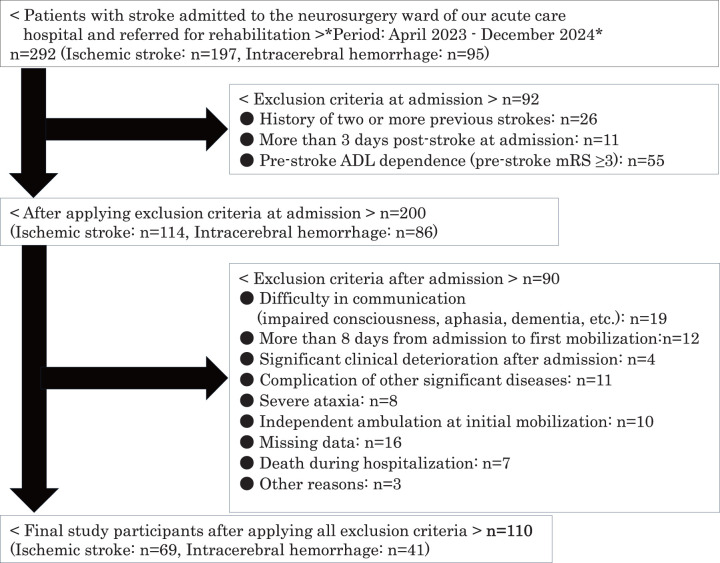
Study participant flow diagram. ADL, activities of daily living; mRS, modified Rankin Scale

Exclusion criteria at admission were a history of previous stroke (n = 26), hospitalization ≥3 days after onset (n = 11), and pre-admission modified Rankin Scale (mRS) score ≥3 (n = 55), totaling 92 exclusions. An mRS score ≤2 was defined as pre-stroke activities of daily living (ADL) independence, and ≥3 as pre-stroke ADL dependence. Exclusion criteria during hospitalization included difficulty communicating due to impaired consciousness, aphasia, or dementia (n = 19); requiring ≥8 days from admission (day 1) to initial mobilization (n = 12); significant clinical deterioration or complications of other diseases (n = 4); complication of other significant diseases (n = 11); marked ataxia (n = 8); independent gait at initial mobilization (n = 10); in-hospital death (n = 7); missing data (n = 16); and other reasons (n = 3), totaling 90 exclusions. Consequently, 110 patients (69 with cerebral infarction and 41 with intracerebral hemorrhage) were included in the analysis (Fig. 1).

This study adhered to the Declaration of Helsinki, with strict measures to protect personal information and maintain anonymity, and was approved by Nasu Red Cross Hospital’s ethics committee (Approval No.: 2025-08).

### Methods

Data collected included age, sex, stroke type, lesion location (right hemisphere, left hemisphere, infratentorial), day of initial physical therapy, day of initial mobilization, day of FACT assessment, length of hospital stay, National Institutes of Health Stroke Scale (NIHSS) score at initial PT, SIAS lower extremity motor function score for the paretic side (SIAS-LE), total FACT score and its subitems 1–10, amount of PT intervention, and Functional Ambulation Categories (FAC) at discharge. All the assessors were PTs. Hospital day 1 was defined as the day of admission.

Assessment timing: Subjects were assessed with the SIAS lower extremity subscale on the day of initial mobilization and with the FACT early after initial mobilization; both assessments were completed within the first 7 days of hospitalization. FAC was assessed at discharge. Decisions regarding mobilization initiation and permissible vital signs for physical therapy were based on the instructions from neurosurgeons and neurologists. The initial mobilization day was defined as the first day the patient stood or was transferred to a wheelchair after admission. The amount of physical therapy intervention was evaluated in units, with 1 unit equivalent to 20 min, according to the Japanese inpatient system.

The NIHSS^19)^ is an internationally used scale for assessing neurological symptoms and severity after stroke. It consists of 11 items (total score 0–42), evaluating consciousness, motor function, and higher brain function. Higher scores indicate greater stroke severity: ≥14 severe, 6–13 moderate, and 0–5 mild^20)^.

The SIAS^11)^ comprehensively evaluates stroke impairment using 22 items (total score 0–76). The lower extremity motor function items assess voluntary movement in hip flexion, knee extension, and ankle dorsiflexion on the paretic side, each scored 0–5 (total 15), with higher scores indicating better paretic lower limb function.

The FACT^12,13)^ assesses trunk control in the sitting position and comprises 10 items scored 1–3, with a total of 20 points per item. Higher scores indicate better trunk control (Table 1).

**Table 1.** Functional Assessment for Control of Trunk (FACT)

Item	Objective (component)	Test method (judgment standard)	Score (total 20 points)
1	Ability to retain static seated position (using upper limb support)	Ability to sit upright for more than 10 seconds when grabbing a railing or seat surface using the upper limbs	Able: 1 points Unable: 0 points
2	Ability to retain static seated position (not using upper limb support)	Ability to sit upright for more than 10 seconds without using the upper limbs	Able: 1 points Unable: 0 points
3	Downward shift in center of gravity/reach; mild trunk rotation accompanied by pro-/anti-gravitational activities	Ability to grab the ankle on the other side using either the left or right hand and then return to the original position	Able: 1 points Unable: 0 points
4	Forward shift in the center of gravity accompanied by resilience of the lower limbs/trunk; further shift to the right and left in the center of gravity accompanied by selective gentle movement of the pelvis/trunk	Ability to move at least 10 cm to both the right and left side while lifting the bilateral buttocks	Able: 2 points Unable: 0 points
5	Wide-range unilateral shift in center of gravity accompanied by resilience	Ability to lift the unilateral buttock from the seat for at least 3 seconds (bilateral)	Bilateral: 2 points Unilateral: 1 points Unable: 0 points
6	Slight backward shift in center of gravity accompanied by resilience; ability to retain the ipsilateral trunk while lifting the unilateral lower limb	Ability to lift the right or left thigh and remain for at least 3 seconds with the sole of the foot not touching the ground (bilateral)	Bilateral: 2 points Unilateral: 1 points Unable: 0 points
7	Wide-range backward shift in center of gravity accompanied by resilience; ability to retain the bilateral trunk while both lower limbs are lifted	Ability to lift both the right and left thighs with both feet not touching the ground for at least 3 seconds	Able: 2 points Unable: 0 points
8	Wide-range lateral shift in center of gravity; further selective rotation of the pelvis and trunk	Ability to lift the buttocks one side at a time and move both forward and backwards with the bottom	Able: 3 points Unable: 0 points
9	Rotation with the trunk stretched	The examiner should touch the seat surface 20 cm posterior to the sacral bone. The examinee should look over their shoulder and say how many fingers the examiner is showing, which should be changed 3 times at 1-second intervals (can imitate the shapes of the hand)	Able: 3 points Unable: 0 points
10	Maximum extension of the spinal column	Ability to raise the right or left upper limb (shoulder joint bending) with maximum effort; ability to raise the humeral bone vertically to the ground at the middle position of the adduction/extorsion of the shoulder joint	Able: 3 points Unable: 0 points

The scale assesses static (items 1 and 2) and dynamic (items 3–10) seated trunk control. Total score: 20 points.

The FAC^21)^ assesses walking independence as follows: 0, non-ambulatory or requires 2-person assistance; 1, requires continuous support; 2, requires intermittent support for balance or assistance; 3, requires supervision on level ground; 4, independent on level ground; and 5, independent on stairs/uneven ground. Higher scores indicate greater walking independence.

For analysis, FAC scores were categorized as follows: 4–5, independent on level ground (Level-ground Independent group); 0–3, dependent on level ground (Level-ground Dependent group); 5, independent on uneven ground (Uneven-ground Independent group); and 0–4, dependent on uneven ground (Uneven-ground Dependent group).

After categorization, survey items were compared between Level-ground Independent and Dependent groups and between Uneven-ground Independent and Dependent groups. Influential FACT subitems for each independent gait level and predictive cutoff values of the total FACT score were investigated. The percentage of patients scoring each FACT subitem per FAC score was also calculated.

### Statistical analysis

Comparisons between independent and dependent groups for both level and uneven ground were performed using univariate analysis, after confirming normality with the Shapiro–Wilk test. Age was analyzed using the unpaired t-test, nominal variables using Fisher’s exact test, and other continuous variables using the Mann–Whitney U test.

Logistic regression analysis (variable reduction method) was performed to identify predictive factors for Level- and Uneven-ground Independent groups. Dependent variables were: (1) independence on level ground (FAC ≥4; Model 1), and (2) independence on uneven ground (FAC = 5; Model 2). Independent variables were selected based on prior reports; age affects trunk control^7)^ and gait^5)^; neurological severity^22)^ and the day of initial mobilization^23–25)^ are important prognostic predictors. Furthermore, the dynamic sitting balance affects gait more than the static sitting balance^3)^. Thus, 11 independent variables were set: age, NIHSS score, day of initial mobilization, and FACT subitems 3–10 (dynamic trunk control).

Multicollinearity was considered acceptable if the variance inflation factor (VIF) ≤5^26)^ and Spearman's rank correlation coefficient between variables <0.9^26)^.

For Models 1 and 2, receiver-operating characteristic (ROC) analysis was used to calculate the area under the curve (AUC), cutoff value, sensitivity, and specificity, with thresholds determined by the Youden index. AUC ≥0.7 indicates moderate accuracy and ≥0.9 indicates high accuracy^26)^; clinical utility was judged for AUC ≥0.7. Analyses were performed using EZR (version 1.68; Saitama Medical Center, Jichi Medical University, Saitama, Japan), with the significance level set at p <0.05.

## Results

Basic characteristics and univariate analysis results are presented in Table 2. The mean age of participants was 69 years. The median day of FACT assessment was hospital day 3, and the median hospital length of stay was 16 days. NIHSS severity distribution was as follows: 54 mild cases and 56 moderate-to-severe cases. FAC scores were distributed as follows: 5 (n = 20), 4 (n = 40), 3 (n = 3), 2 (n = 16), 1 (n = 26), and 0 (n = 5). Comparisons between independent and dependent groups revealed significant differences in age, stroke type, day of initial mobilization, NIHSS, total FACT score, amount of physical therapy intervention, and discharge destination.

**Table 2.** Participant characteristics and univariate analysis results

Item	All (n = 110)	Level-ground Independent Group FAC: 4–5 (n = 60)	Level-ground Dependent Group FAC: 0–3 (n = 50)	p-Value	Uneven-ground Independent Group FAC: 5 (n = 20)	Uneven-ground Dependent Group FAC: 0–4 (n = 90)	p-Value
Demographics							
Age (years)	68.7 ± 12.6	66.6 ± 13.7	72.7 ± 9.5	0.009	61.3 ± 14.5	70.4 ± 11.5	0.003
Sex, male	78 (70.9)	45 (75.0)	33 (66.0)	0.399	14 (70.0)	64 (71.1)	>0.900
Stroke characteristics							
Type, cerebral infarction	69 (62.7)	45 (75.0)	24 (48.0)	0.005	15 (75.0)	54 (60.0)	0.307
Lesion, right/left hemisphere	47/52 (42.7/47.3)	29 (48.3)	18 (36.0)	0.054	11 (55.0)	36 (40.0)	0.059
Clinical timeline (days)							
Day of initial physical therapy session	2 [2, 2]	2 [2, 2]	2 [2, 2]	0.963	2 [1, 2]	2 [2, 2]	0.873
Day of initial mobilization	2 [2, 3]	2 [2, 3]	3 [2, 4]	0.001	2 [1, 3]	2 [2, 4]	0.029
Day of FACT assessment	3 [2, 5]	3 [2, 5]	3 [2, 4]	0.791	4 [2, 5]	3 [2, 4]	0.173
Length of hospital stay	16 [13, 24]	15 [12, 21]	20 [15, 26]	0.002	13 [12, 15]	19 [14, 25]	<0.001
Assessment scores							
NIHSS score	6 [2, 12]	2.5 [1, 4]	13 [8, 20]	<0.001	1 [1, 2]	8 [4, 14]	<0.001
Mild 0–5	54 (49.1)						
Moderate 6–13	33 (30.0)						
Severe 14	23 (20.9)						
SIAS lower extremity score	12 [5, 15]	12 [8, 15]	12 [3, 15]	0.153	12 [6, 15]	12 [6, 15]	0.874
FACT total score	13 [3, 17]	16 [13, 20]	3 [0, 9]	<0.001	18.5 [17, 20]	10 [2, 14]	<0.001
Intervention and outcome							
Physical therapy dose(unit)/day *1 unit = 20 min	1.1 [0.8, 1.5]	0.8 [0.5, 1.2]	1.4 [1.1, 1.6]	<0.001	0.6 [0.4, 0.8]	1.3 [0.8, 1.5]	<0.001
Discharge to home	28 (25.5)	28 (46.7)	0 (0.0)	<0.001	18 (90.0)	10 (11.1)	<0.001
FAC							
Score 0	5 (4.6)						
Score 1	26 (23.6)						
Score 2	16 (14.5)						
Score 3	3 (2.7)						
Score 4	40 (36.4)						
Score 5	20 (18.2)						

Data are mean ± SD, median [IQR], or n (%). p-Values: unpaired t-test (age), Fisher’s exact test (categorical), or Mann–Whitney U test (other continuous). Groups: Level-ground Independent (FAC 4 and 5), Dependent (FAC 0–3); Uneven-ground Independent (FAC 5), Dependent (FAC 0–4).

FAC, Functional Ambulation Categories; FACT, Functional Assessment for Control of Trunk; NIHSS, National Institutes of Health Stroke Scale; SIAS, Stroke Impairment Assessment Set; SD, standard deviation; IQR, interquartile range

Logistic regression analysis showed all inter-variable correlation coefficients were <0.9 (Table 3), and no variable had a VIF ≥5, indicating no multicollinearity. Selected independent variables were as follows: for Model 1 (level-ground independence), FACT subitems 4 and 5 and NIHSS (odds ratios [OR]: 4.71, 95% confidence interval [CI]: 1.36–16.3; 2.89, 95% CI: 1.08–7.72; 0.67, 95% CI: 0.54–0.83); for Model 2 (uneven-ground independence), FACT subitem 8 and NIHSS (OR: 1.87, 95% CI: 1.19–2.94; 0.56, 95% CI: 0.38–0.82) (Table 4).

**Table 3.** Correlation coefficients among assessment tools

Variable	Age	FAC score	FACT total score	Day of initial mobilization	NIHSS score	FACT item 3	FACT item 4	FACT item 5	FACT item 6	FACT item 7	FACT item 8	FACT item 9	FACT item 10
Age	1	−0.284^*^	−0.396^*^	−0.042	0.179	−0.125	−0.242^*^	−0.328^*^	−0.230^*^	−0.337^*^	−0.393^*^	−0.366^*^	−0.172
FAC score		1	0.859^*^	−0.378^*^	−0.854^*^	0.646^*^	0.766^*^	0.733^*^	0.777^*^	0.699^*^	0.569^*^	0.541^*^	0.806^*^
FACT total score			1	−0.326^*^	−0.797^*^	0.738^*^	0.804^*^	0.827^*^	0.841^*^	0.808^*^	0.724^*^	0.712^*^	0.806^*^
Day of initial mobilization				1	0.386^*^	−0.409^*^	−0.361^*^	−0.317^*^	−0.389^*^	−0.278^*^	−0.067	−0.137	−0.396^*^
NIHSS score					1	−0.69^*^	−0.718^*^	−0.696^*^	−0.755^*^	−0.671^*^	−0.503^*^	−0.458^*^	−0.754^*^
FACT item 3						1	0.798^*^	0.646^*^	0.809^*^	0.577^*^	0.317^*^	0.333^*^	0.798^*^
FACT item 4							1	0.678^*^	0.862^*^	0.723^*^	0.398^*^	0.417^*^	0.794^*^
FACT item 5								1	0.724^*^	0.603^*^	0.643^*^	0.390^*^	0.723^*^
FACT item 6									1	0.769^*^	0.423^*^	0.444^*^	0.833^*^
FACT item 7										1	0.423^*^	0.536^*^	0.646^*^
FACT item 8											1	0.474^*^	0.398^*^
FACT item 9												1	0.417^*^
FACT item 10													1

Spearman’s rank correlation coefficients (r) are shown. ^*^p <0.01.

FAC, Functional Ambulation Categories; FACT, Functional Assessment for Control of Trunk; NIHSS, National Institutes of Health Stroke Scale

**Table 4.** Results of multivariable logistic regression analysis (backward selection method)

	Variable	β-coefficient	OR	95% CI	p-Value
Model 1					
Level-ground Independent Group (FAC 4 and 5)	FACT item 4	1.54968	4.71	1.36–16.3	0.014
	FACT item 5	1.06125	2.89	1.08–7.72	0.035
	NIHSS	−0.40047	0.67	0.54–0.83	<0.001
Model 2					
Uneven-ground Independent Group (FAC5)	FACT item 8	0.62593	1.87	1.19–2.94	0.006
	NIHSS	−0.57981	0.56	0.38–0.82	0.003

Model 1: Level-ground Independence (FAC 4 and 5); Model 2: Uneven-ground Independence (FAC 5); OR, odds ratio; CI, confidence interval; FAC, Functional Ambulation Categories; FACT, Functional Assessment for Control of Trunk; NIHSS, National Institutes of Health Stroke Scale

**Fig. 2.**
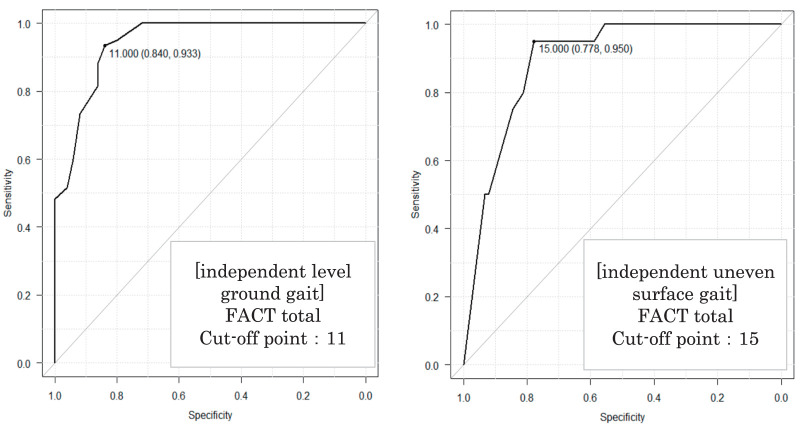
ROC curves for FACT total score. Level-ground independence (FAC ≥4). Uneven-ground independence (FAC = 5). FACT, Functional Assessment for Control of Trunk; ROC, receiver-operating characteristic; FAC, Functional Ambulation Categories

ROC curve analysis for predicting independent gait at discharge using the total FACT score yielded cutoff values of 11 points for level-ground (sensitivity: 84.0%; specificity: 93.3%; AUC: 0.95; 95% CI: 0.91–0.99) and 15 points for uneven-ground (sensitivity: 77.8%; specificity: 95.0%; AUC: 0.89; 95% CI: 0.83–0.96). The AUC values indicated moderate-to-high accuracy (Table 5 and Fig. 2).

**Table 5.** Predictive accuracy of FACT total score for gait independence (ROC analysis)

	Cutoff point	Sensitivity (%)	Specificity (%)	AUC	95% CI
Model 1					
Level-ground Independent Group (FAC 4 and 5)	11.0	84.0	93.3	0.95	0.91–0.99
Model 2					
Uneven-ground Independent Group (FAC 5)	15.0	77.8	95.0	0.89	0.83–0.96

FACT, Functional Assessment for Control of Trunk; ROC, receiver-operating characteristic; AUC, area under the ROC curve; CI, confidence interval; FAC, Functional Ambulation Categories

The percentage of patients scoring points for each FACT subitem per FAC score is shown in Table 6. Comparing FAC 5 vs. FAC 4, the FAC 5 group had higher scores for item 5 (bilaterally possible) and items 8 and 9. Comparing FAC 4 vs. FAC 3, the FAC 3 group had lower scores on items 5 (bilaterally possible), 8, and 10 but a higher percentage on item 7. Comparing FAC 3 vs. FAC 2, the FAC 2 group scored lower on items 3, 4, 5, 6, 7, and 9. Comparing FAC 2 vs. FAC 1, the FAC 1 group had lower percentages of scores on most items. None of the patients with an FAC of 0 scored any of the subitems.

**Table 6.** Proportion of participants scoring on FACT subitems by FAC

FAC score	n	Item 1 (%)	Item 2 (%)	Item 3 (%)	Item 4 (%)	Item 5 (%)	Item 6 (%)	Item 7 (%)	Item 8 (%)	Item 9 (%)	Item 10 (%)
						(bilateral/unilateral)	(bilateral/unilateral)				
5	20	100	100	100	100	90	100	90	75	70	100
						(85/5)	(100/0)				
4	40	100	100	100	97	90	95	77	28	33	100
						(57/33)	(92/3)				
3	3	100	100	100	100	100	100	100	0	33	67
						(0/100)	(100/0)				
2	16	100	100	87	56	62	81	31	6	6	69
						(19/43)	(56/25)				
1	26	69	53	37	12	8	23	0	0	0	4
						(0/8)	(4/19)				
0	5	0	0	0	0	0	0	0	0	0	0
						(0/0)	(0/0)				

Data are the proportion of participants scoring >0 on each subitem. For items 5 and 6, proportions for bilateral/unilateral performance are in parentheses.

FACT, Functional Assessment for Control of Trunk; FAC, Functional Ambulation Categories; n, number

## Discussion

This study aimed to identify predictive FACT subitems and cutoff values for independent gait at discharge in patients with acute stroke.

The results showed that for FAC ≥4 (level-ground independence), FACT items 4 (“Ability to move at least 10 cm to both the right and left side while lifting the bilateral buttocks”) and 5 (“Ability to lift the unilateral buttock from the seat for at least 3 seconds [bilateral]”) were selected as predictors. For FAC = 5 (uneven-ground independence), FACT item 8 (“Ability to lift the buttocks one side at a time and move both forward and backwards with the bottom”) was selected.

The cutoff values of the total FACT score for predicting independent gait at discharge were 11 points for level ground and 15 points for uneven ground, with the AUC results suggesting clinical utility.

These findings suggest that early trunk control assessment using the FACT is a clinically useful scale for predicting gait prognosis in patients with acute stroke with high accuracy.

### Univariate analysis results (Level-ground and Uneven-ground Groups)

Compared with independent groups, dependent groups were older, had more hemorrhagic strokes, higher NIHSS scores, and lower total FACT scores. Furthermore, initial mobilization was delayed (interquartile range: 2–4 days), the amount of physical therapy intervention was greater, hospital stays were longer, and more patients were transferred to convalescent rehabilitation wards, with significant differences in these items. No significant difference was found in SIAS-LE scores.

Previous studies have reported better functional outcomes in younger patients^27)^ and those with lower NIHSS scores^28,29)^. Patients with hemorrhagic stroke tend to have higher NIHSS scores on admission^30)^, and higher NIHSS scores are associated with worse trunk control^6)^. Earlier initial mobilization (typically within 48–72 h after the first 24 h) is associated with better functional outcomes^23–25)^. Studies have also reported that trunk control influences gait ability more than paretic limb function^6)^. The significantly better trunk control in both independent groups and the lack of a significant difference in paretic lower limb function support these previous findings.

Thus, compared to independent groups, dependent groups, despite similar paretic limb function, tended to be neurologically more severe and to have worse trunk control, suggesting a potential association with poorer gait ability, which may have necessitated more physical therapy intervention, longer hospital stays, and more frequent transfers to convalescent rehabilitation hospitals.

### Multivariate analysis results for Models 1 and 2 (selection of NIHSS, non-selection of age and initial mobilization day)

The selection of the NIHSS aligns with previous reports, identifying it as an important prognostic predictor of gait^22)^.

The non-selection of age might be because age is known to affect trunk control^7)^ and gait^5)^, and trunk control affects ADL^31)^. This sample excluded older adults with pre-stroke ADL dependence (mRS ≥3)^32)^, making the overall sample younger^6,17,18)^ than those in previous studies. Consequently, the patients were likely ADL-independent with good pre-stroke trunk control and gait ability, minimizing the impact of age-related functional differences.

The non-selection of the initial mobilization day might be because the NIHSS and FACT subitems (assessing dynamic sitting balance early after mobilization) had a stronger influence on gait ability at discharge.

### Multivariate analysis results for Model 1 (selection of items 4 and 5)

Item 4 (“Ability to move at least 10 cm to both the right and left side while lifting the bilateral buttocks”): The design and characteristics of this task suggest it assesses elements similar to sit-to-stand/stand-to-sit and lateral weight-shifting ability^12,13)^. Both of these elements, the former^33)^ and the latter^3,4,34–37)^, are known to influence gait. Item 4 is likely a complex, high-difficulty task that integrates these elements to evaluate coordinated lower limb and trunk movement during forward and lateral weight shifts. Therefore, it was selected as a predictor because it was suggested to have a high impact on gait ability.

Item 5 (“Ability to lift the unilateral buttock from the seat for at least 3 seconds (bilateral)”): The task's design and characteristics suggest that it assesses extensive lateral weight-shifting ability^12,13)^. Stroke patients often experience functional decline in the lateral abdominal muscles (e.g., external oblique on the paretic side), affecting trunk lateral flexion^34)^, leading to pelvic obliquity, impaired lateral sitting balance^35)^, and reduced supportability of the paretic limb^36)^. Furthermore, balance impairment is more strongly related to lateral than to anteroposterior weight-shifting ability^37)^. Thus, the extensive lateral weight-shifting ability in item 5, often impaired post-stroke, significantly affects balance and paretic limb support while standing. Therefore, it was suggested that item 5 contributes more to balance function than items 3, 6, and 7 (which primarily assess anteroposterior or mild lateral shifting), leading to its selection in the model.

Based on subacute samples, the dynamic sitting item of the TIS, somewhat similar to FACT item 5, has been reported to influence gait ability and independence more than other TIS items^3,4)^, supporting our results.

In summary, coordinated movement of the lower limbs and trunk during forward and lateral weight shifts and extensive lateral weight-shifting ability appear crucial for independent gait on level ground.

### Multivariate analysis results for Model 2 (selection of item 8)

Item 8 (“Ability to lift the buttocks one side at a time and move both forward and backward with the bottom”): The task’s design and characteristics suggest that it assesses rotational ability of the bilateral lower trunk during extensive lateral weight shifts^12,13)^. The importance of lateral weight shifting for gait has been reported^3,4, 34 –37)^. Trunk rotation also contributes to gait^38,39)^. However, the TIS coordination item, similar to FACT item 8, did not correlate with achieving supervised walking (FAC ≥3) on level ground in subacute patients^4)^.

In this study, the scoring rate for item 8 was approximately 70% in cases with FAC = 5, but only approximately 30% in cases with FAC = 4, and it was not selected in Model 1 multivariate analysis. These results support previous research findings that trunk rotation may not correlate with level-ground gait^4)^. The high difficulty of item 8 likely explains this. First, in addition to being performed in a sitting position where posterior pelvic tilt is common^40)^, stroke patients are more prone to posterior pelvic tilt due to abdominal muscle weakness^1)^. This makes trunk extension and anterior pelvic tilt, important for rotation, difficult^41)^, resulting in a task that challenges trunk rotation.

Item 8 also requires extensive lateral weight-shifting ability, assessed in item 5 bilaterally during the butt-walking process, demanding even greater proficiency than item 5. This is suggested by the higher bilateral scoring rate for item 5 in the FAC 5 group (85%) vs. the FAC 4 group (57%). The scoring rate for item 8 itself was also lower (<30% in FAC = 4 vs. ~70% in FAC = 5) than for the other items, confirming its higher difficulty.

The ability to perform this challenging task likely reflects trunk rotation capability exceeding that required for level-ground walking, potentially associated with uneven-ground walking ability. Studies reported that trunk rotation may relate to endurance, adaptation to external environments, and complex walking tasks (e.g., dual-task walking)^42)^. Therefore, rotational ability of the bilateral lower trunk during extensive lateral weight shifts, as assessed by item 8, though not essential for level-ground walking, may be necessary for independent gait on uneven ground, explaining its selection as a predictor.

Thus, the rotational ability of the bilateral lower trunk during extensive lateral weight shifts appears to be crucial for independent gait on uneven ground.

### Study limitations

This study had several limitations.

First, the study employed a single-center retrospective design with a limited sample size of 110. Consequently, for the logistic regression model that used 11 independent variables, the number of samples relative to the events of independent gait on level and uneven ground was small^26)^, and the risk of overfitting cannot be ruled out. Furthermore, the adjusted confounders were limited to age, neurological severity, and the initial day of mobilization. Unadjusted factors or those related to the exclusion criteria (e.g., communication difficulties and severe higher brain dysfunction) may remain as residual confounders, necessitating caution regarding internal validity.

Second, its external validity is limited. The sample was relatively young and was from a single center; therefore, generalizing the results to all patients with acute stroke requires caution. Specifically, the mean age of our cohort (69 years) is younger than that often reported in typical stroke populations^6,17,18)^. Although our small sample size precluded a separate analysis of older subgroups, advancing age has been reported to be associated with declines in trunk control and gait ability^5,7)^, suggesting that the predictive validity of our findings might differ in this population. Therefore, the applicability of our results to older stroke patients should be viewed with caution, necessitating future studies with larger, more age-diverse samples that include subgroup analyses by age. Cross-validation was not performed, and reproducibility in other settings and patient populations requires further investigation.

Third, this study had methodological limitations. Quantitative assessments of trunk range of motion or muscle activity were not performed, potentially influencing the FACT scores. The time required to achieve gait independence was not analyzed. Additionally, we did not perform subgroup analyses based on NIHSS severity levels due to the limited sample size within subgroups. Given that a higher NIHSS score may negatively impact both trunk function and gait ability^6,22)^, it is plausible that the predictive factors for gait independence might also differ by severity. Therefore, future research should explore whether the predictive FACT cutoff values and/or subitems differ between patients with mild vs. moderate-to-severe strokes. Furthermore, while we controlled for several factors, the potential influence of unmeasured confounders related to the exclusion criteria (e.g., communication difficulties and severe higher brain dysfunction) cannot be ruled out. Additionally, to more accurately isolate and compare the predictive power of the FACT subitems themselves, future studies would benefit from employing methods such as propensity score matching^26)^ to ensure comparability between groups, which was not feasible in this study due to the limited sample size.

Therefore, these results are preliminary and should be interpreted with caution. Future prospective multicenter studies with larger sample sizes are required for further validation. Addressing these issues is crucial for the efficient development of physical therapy for trunk control and gait acquisition in patients with acute stroke.

## Conclusions

This study identified predictive FACT subitems assessed early after mobilization and their cutoff values for independent gait at discharge in patients with acute stroke (median FACT assessment: hospital day 3; median hospital stay: 16 days).

For level-ground independent gait, coordinated movement of the lower limbs and trunk during forward and lateral weight shifts and extensive lateral weight-shifting ability (items 4 and 5) were key predictors. For uneven-ground independent gait, rotational ability of the bilateral lower trunk during extensive lateral weight shifts (item 8) was crucial.

The cutoff values for the total FACT score were 11 points for level ground and 15 points for uneven ground.

The significance of this study lies in identifying specific subitems and cutoff values using the FACT, a scale useful for both assessment and exercise therapy, to predict the functional outcome of independent gait in the acute stroke phase. These findings may help objectively determine the priority of trunk assessment and intervention for gait acquisition early after stroke onset and support planning of appropriate rehabilitation strategies and discharge planning.
